# FoxO3a confers cetuximab resistance in RAS wild-type metastatic colorectal cancer through c-Myc

**DOI:** 10.18632/oncotarget.13105

**Published:** 2016-11-04

**Authors:** Yiyi Yu, Mengzhou Guo, Ye Wei, Shan Yu, Hong Li, Yan Wang, Xiaojing Xu, Yuehong Cui, Jiawen Tian, Li Liang, Ke Peng, Tianshu Liu

**Affiliations:** ^1^ Department of Medical Oncology, Zhong Shan Hospital, Fu Dan University, Shanghai, China; ^2^ Department of General Surgery, Zhong Shan Hospital, Fu Dan University, Shanghai, China

**Keywords:** cetuximab resistance, FoxO3a, c-Myc, RAS, colorectal cancer

## Abstract

Resistance to epidermal growth factor receptor (EGFR) targeted monoclonal antibody therapy represents a clinical challenge in patients suffered from RAS wild-type (WT) metastatic colorectal cancer (mCRC). However, the molecular mechanisms and key factors conferring this resistance are largely unknown. Forkhead transcription factors of the O class 3a (FoxO3a), an important regulator of cell survival, has been reported with dual functions in tumor recently. In this study, we found that FoxO3a was highly expressed in cetuximab resistant CRC tissues compared with cetuximab sensitive tissues. We therefore further analyzed its function in induced cetuximab resistant RAS-WT CRC cells (Caco2-CR) and intrinsic resistant cells with BRAF mutation (HT29). We found that FoxO3a was significantly up-regulated in Caco2-CR as well as in cetuximab treated HT29 cells. Knockdown of FoxO3a could sensitize these cells to cetuximab treatment with reduced cell proliferation and migration ability. Further, biochemical experiments demonstrated that FoxO3a directly bind to c-Myc promoter and activated the transcription of the c-Myc gene, thus participated in regulating of c-Myc downstream genes, including ACO2, LARS2, MRPL12 and PKM2 in these resistant cells. Moreover, knockdown of c-Myc elevated cell apoptosis to cetuximab treatment and suppressed cell proliferation and migration ability consistently. Altogether, our study indicates that FoxO3a might be a key regulator in cetuximab resistance through up-regulating c-Myc in colorectal cancer targeted therapy.

## INTRODUCTION

Metastatic colorectal cancer (mCRC) as one of the most common causes of cancer-related deaths has attracted many researchers' attention in recent years. The anti-epidermal growth factor receptor (EGFR) antibody, cetuximab, is used to treat RAS wild type mCRC [1–3]. However, clinical data indicate that even the best responses were transient and eventually all patients developed acquired resistance which triggered intensive interests on the molecular mechanisms of primary and acquired resistance to cetuximab or cetuximab combined with chemotherapeutics. Though alterations in RAS and other genes are found in primary and secondary resistance to anti-EGFR antibody therapy, mechanisms of resistance to EGFR blockade are far more from clear [4]. Elucidating the factors involved in resistance to anti-EGFR antibody therapy is extremely urgent for effective therapy in mCRC.

FoxO3a is a highly evolutionarily conserved transcription factor. It has been intensively studied for its important roles in programmed cell death, cell cycle regulation, DNA damage repair, vascular development, reactive oxygen species detoxification pathways, longevity and regulation of the innate and adaptive immune responses [5–11]. However, the functions and underlying mechanisms of FoxO3a in cancer are still under investigation. Previous studies found that FoxO3a participated apoptotic pathway and was considered as a tumor suppressor gene [12, 13]. Bullock et al. found decreased expression of FoxO3a within human colorectal cancer tissues usually associated with advanced recurrence and poor survival [14], suggesting that FoxO3a might act as a tumor suppressor in colorectal cancer. Our group also found that FoxO3a are associated with gastric cancer prognosis [15]. However, recent literatures revealed that FoxO3a also have pro-oncogenic functions. FoxO3a promotes colorectal cancer progression through co-regulation of metastasis relevant genes with beta-catenin [16]. Moreover, suppression of FoxO3a in glioma cells enhanced response to radiotherapy indicating that FoxO3a is involved in radiotherapy resistance [17]. These studies suggest that Foxo3a might play important functions in tumor progression and therapy.

As a transcriptional factor, the function of FoxO3a is highly relied on down stream genes. Researchers have showed a functional association of FoxO3a and c-Myc during cell proliferation and invasion [18, 19]. More, c-Myc is the central factor of cell survival and metabolism regulating a panel of genes in cells [20]. FoxO3a has also been reported regulates reactive oxygen metabolism by inhibiting mitochondrial gene expression [21]. Despite these findings, the association and molecular mechanisms of FoxO3a and c-Myc in anti-EGFR monoclonal antibody therapy resistance are unknown.

Hence, in this paper, we studied the function of FoxO3a in CRC cetuximab resistance with induced cetuximab resistant CRC cells, potential intrinsic resistant cells with BRAF mutation and tumor tissues. We have found that FoxO3a participate in cetuximab resistant through directly binding to c-Myc promoter and activate its transcription, thus promote c-Myc down stream ACO2, LARS2 and MRPL12 genes in RAS WT CRC cells. Knockdown of c-Myc reversed the cetuximab resistance in CRC cells. Our study revealed a novel function and mechanism of FoxO3a in mCRC resistant to cetuximab.

## RESULTS

### Up-regulated expression of FoxO3a in cetuximab resistant CRC tissues and cells

**Figure 1:**
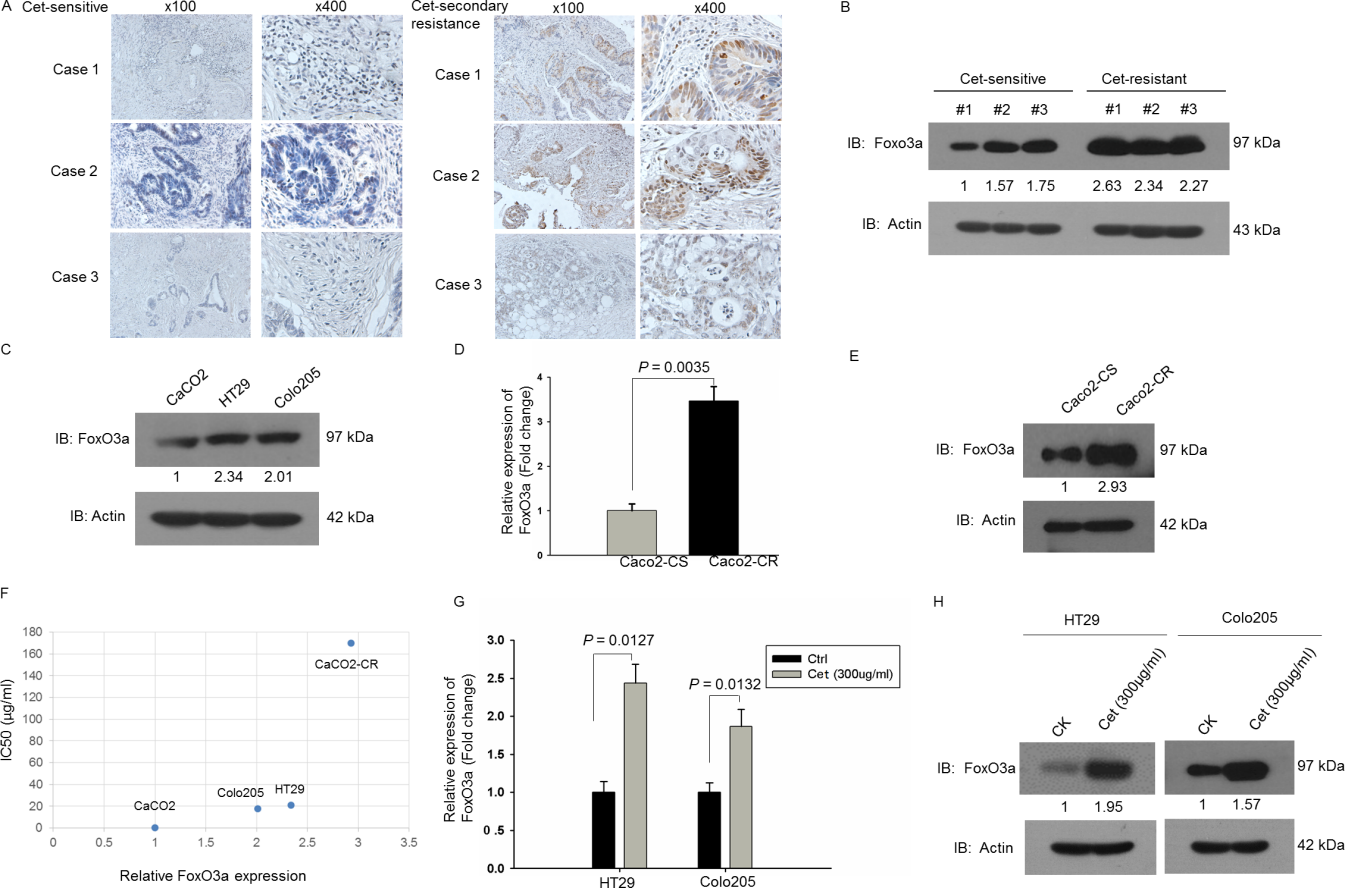
Up-regulated FoxO3a expression in cetuximab resistant RAS WT CRC (**A**) IHC analysis of FoxO3a expression in cetuximab sensitive and resistant CRC tissues. (**B**) Western blotting analysis of FoxO3a protein levels in cetuximab sensitive and resistant CRC tissues. (**C**) Western blotting analysis of FoxO3a protein levels in CRC cells, Caco2, HT29 and Colo205. (**D**) RT-PCR analysis of FoxO3a mRNA levels in parental cetuximab sensitive Caco2-CS and induced cetuximab resistant cells Caco2-CR. (**E**) Western blotting analysis of FoxO3a protein levels in Caco2-CS and Caco2-CR. (**F**) Association of FoxO3a protein levels with IC50. (**G**) RT-PCR analysis of FoxO3a mRNA levels in 300 μg/ml cetuximab treated HT29 and Colo205 cells. (**H**) Western blotting analysis of FoxO3a protein levels in 300 μg/ml cetuximab treated HT29 and Colo205 cells.

We collected and examined FoxO3a expression in cetuximab sensitive or secondary resistant CRC tissues. The IHC results revealed that FoxO3a was highly expressed in cetuximab secondary resistant CRC tissues while FoxO3a was low in cetuximab sensitive CRC tissues (Figure 1A). The western blotting analysis also showed that FoxO3a was highly expressed in cetuximab secondary resistant CRC tissues compared to sensitive counterparts (Figure 1B). And the western blotting analysis of CRC cell lines (cetuximab sensitive: Caco2; cetuximab resistant: HT29, Colo205) showed that expression of FoxO3a was lower in Caco2 compared with HT29 and colo205 cells suggest that FoxO3a might associated with cetuximab resistance (Figure 1C). We then established acquired cetuximab resistant Caco2 cells by stepwise exposure to increasing doses of cetuximab. The growth curve examined by CCK8 (Supplementary Figure S1) and IC50 analysis verified the successful induction of cetuximab resistant cells, Caco2-CR. RT-PCR and western blotting analysis of cetuximab sensitive Caco2 (Caco2-CS) and derived cells with acquired resistant cells (Caco2-CR) revealed the significant upregulation of FoxO3a in Caco2-CR cells (Figure 1D and 1E). Moreover, the expression level of FoxO3a in CRC cells were positively associated with cetuximab IC50 (Figure 1F). Interestingly, FoxO3a was significantly elevated after treated with cetuximab (300 μg/ml) in HT29 and colo205 cells (Figure 1G and 1H). Altogether, our results indicate that FoxO3a is significantly up-regulated in cetuximab resistant RAS WT CRC cells and tissues.

### Knockdown of FoxO3a sensitized CRC cells to cetuximab treatment and reduced cell proliferation and migration ability

**Figure 2:**
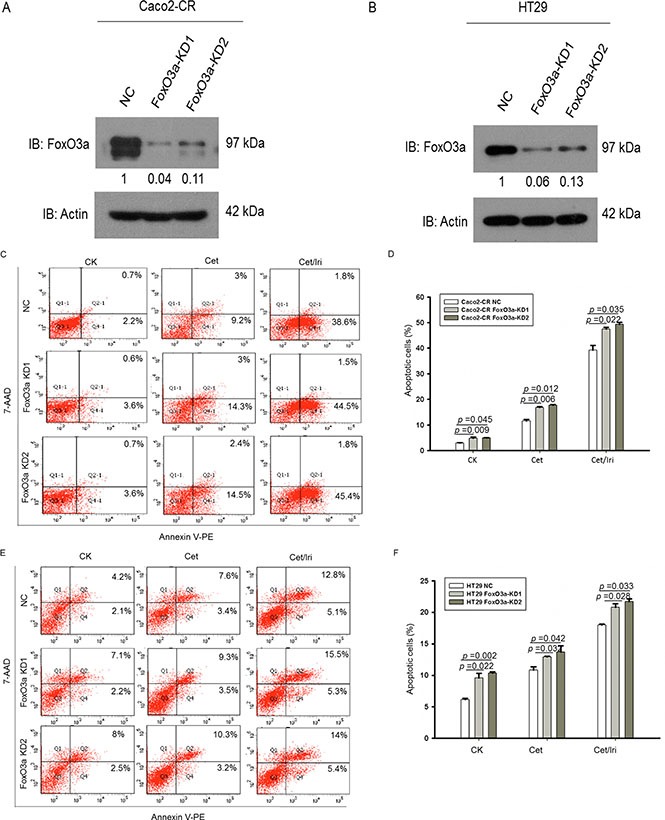
Knockdown of FoxO3a sensitized CRC-CR cells to cetuximab treatment (**A**) Western blotting analysis of FoxO3a protein levels in Caco2-CR cells with FoxO3a knockdown sequences or negative control (NC). (**B**) Western blotting analysis of FoxO3a protein levels in HT29 cells with FoxO3a knockdown sequences or negative control (NC). (**C**, **D**) FACS analysis of cell apoptosis in Caco2-CR cells with or without FoxO3a knockdown treated with cetuximab or combined with irinotecan. (**E**, **F**) FACS analysis of cell apoptosis in HT29 cells with or without FoxO3a knockdown treated with cetuximab or combined with irinotecan.

**Figure 3:**
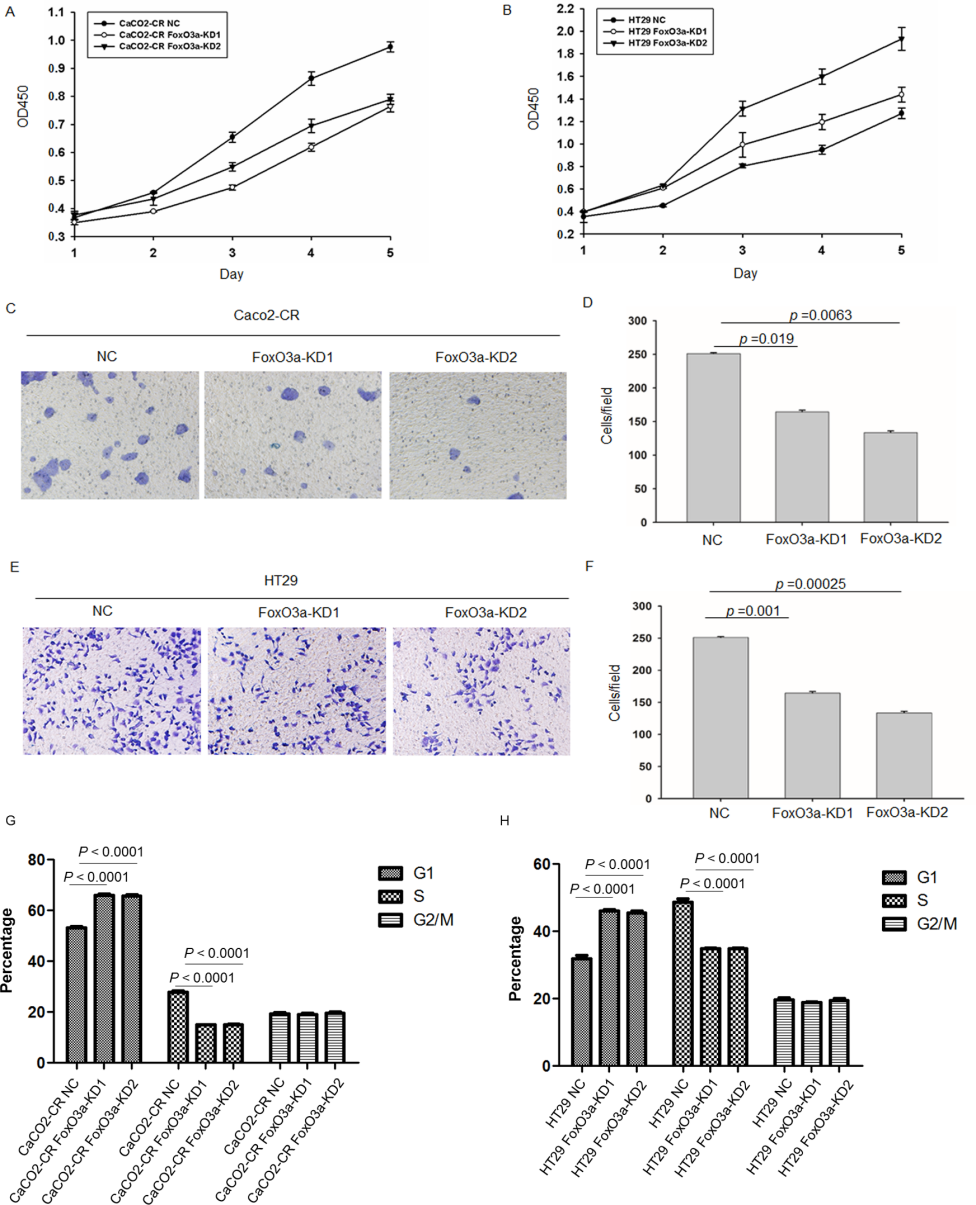
Knockdown of FoxO3a inhibited cell proliferation and migration ability in cetuximab resistant CRC cells (**A**) CCK8 analysis of Caco2-CR cells proliferation ability with FoxO3a knockdown sequences or negative control (NC). (**B**) CCK8 analysis of Caco2-CHT29 cells proliferation ability with FoxO3a knockdown sequences or negative control (NC). (**C**, **D**) Transwell analysis of cell migration ability in Caco2-CR cells with or without FoxO3a knockdown. (**E**, **F**) Transwell analysis of cell migration ability in HT29 cells with or without FoxO3a knockdown.(**G**) FACS analysis of cell cycle in Caco2-CR cells with or without FoxO3a knockdown. (**H**) FACS analysis of cell cycles in HT29 cells with or without FoxO3a knockdown.

In order to investigate the function of FoxO3a in cetuximab resistance, we stably knockdown FoxO3a using lentivirus-mediated shRNA. Western blot verified the efficient knockdown of FoxO3a in Caco2-CR and HT29 cells (Figure 2A and 2B). Apoptotic analysis using annexin-V/7-AAD showed that knockdown of FoxO3a increased cell apoptosis after cetuximab treatment alone or combined with irinotecan (Figure 2C–2F). Further, CCK8 analysis showed that FoxO3a knockdown reduced cell proliferation in Caco2-CR and HT29 cells (Figure 3A and 3B). The percentage of G1 phase cells were significantly increased after FoxO3a suppression while the S phase proportion was decreased (Figure 3G and 3H).Transwell analysis indicated that inhibition of FoxO3a also suppressed cell migration in Caco2-CR and HT29 cells (Figure 3C–3F).

**Figure 4:**
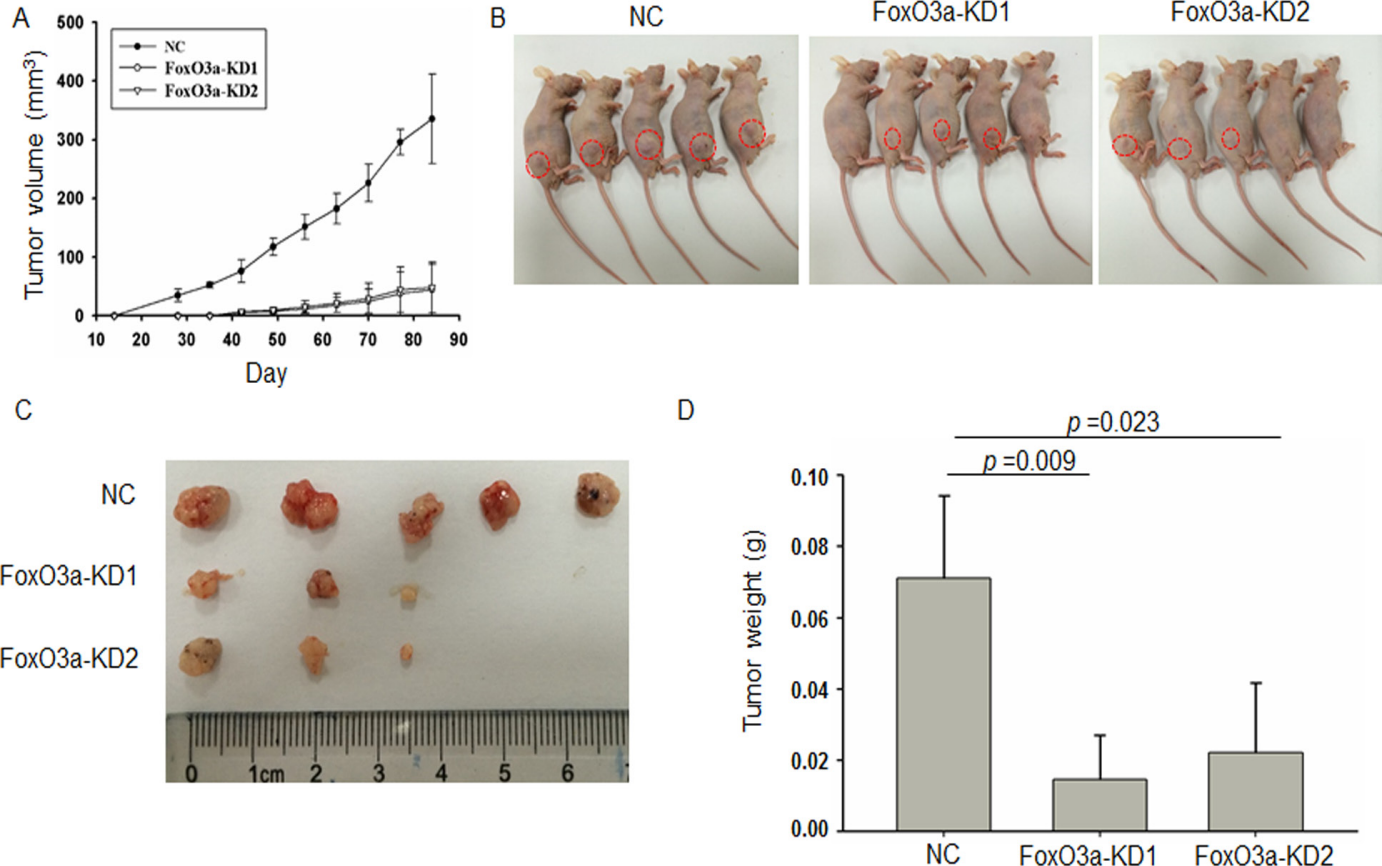
Knockdown of FoxO3a reduced tumorigenesis and growth in CRC animal model (**A**) The volume of tumor formed from subcutaneously inoculated FoxO3a knockdown or negative control (NC) cells into nude mice. (**B**) Photograph of the orthotropic CRC animal models. (**C**) Photograph of the orthotropic CRC tumors. (**D**) The tumor weight of tumor formed from subcutaneously inoculated FoxO3a knockdown or negative control (NC) cells into nude mice.

We further inoculated Caco2-CR cells with or without FoxO3a knockdown in subcutaneous xenograft animal model. Both the tumor volume and weight were significantly decreased in FoxO3a knockdown group compared with NC group (Figure 4). Interestingly, the tumorigenesis ability also reduced in FoxO3a knockdown group compared with NC group (Figure 4C). Taken together, our data suggest that FoxO3a plays important roles in cell proliferation, survival and migration in cetuximab resistant CRC.

### FoxO3a regulates c-Myc function through directly binding to c-Myc promoter and activate c-Myc transcription in cetuximab resistant CRC cells

**Figure 5:**
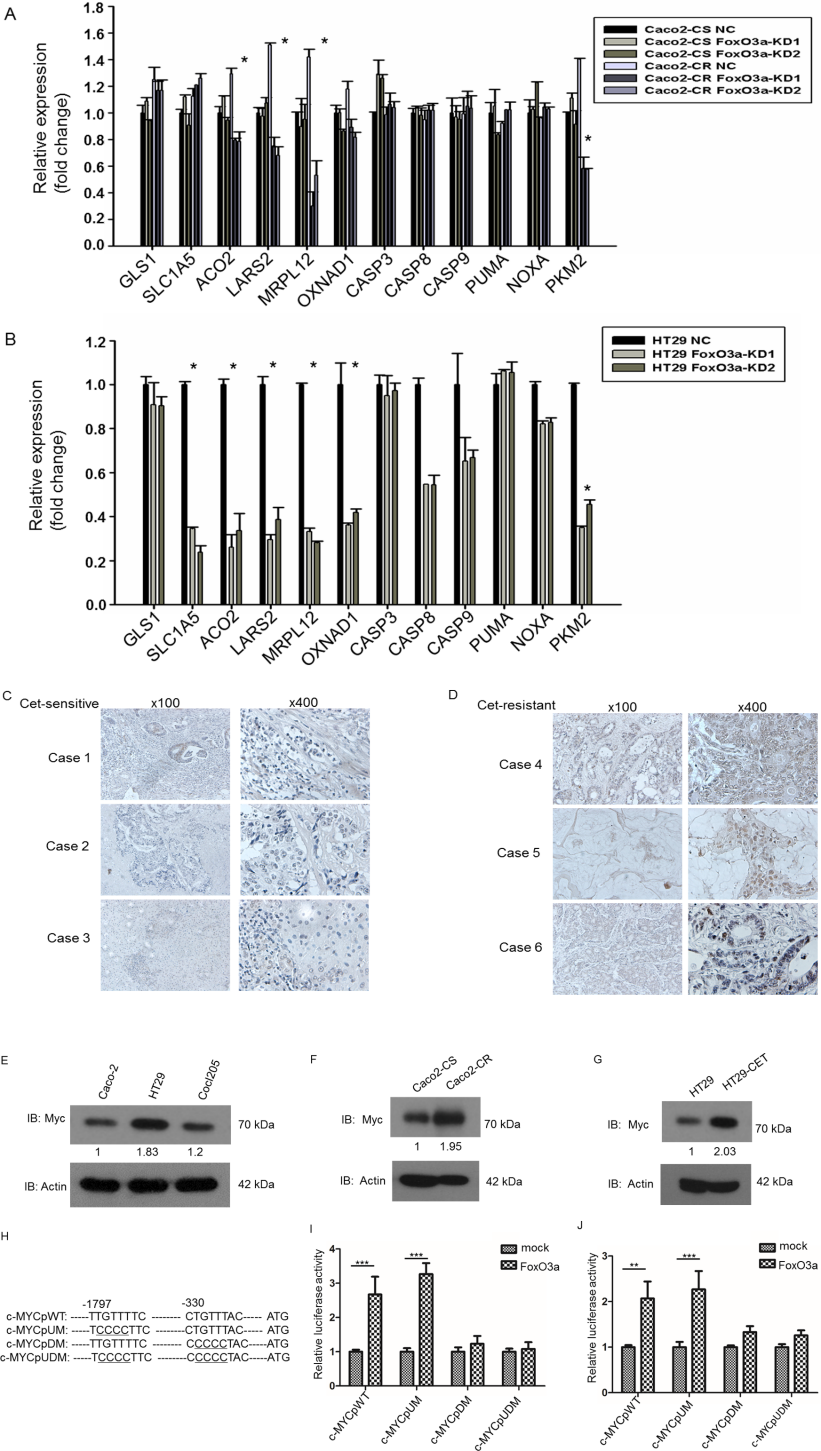
FoxO3a knockdown reduced metabolic genes expression in cetuximab resistant CRC cells and FoxO3a interacted with c-Myc in cetuximab resistant CRC cells (**A**) RT-PCR analysis of metabolic and apoptosis genes in Caco2-CS and Caco2-CR cells with FoxO3a knockdown sequences or negative control (NC). (**B**) RT-PCR analysis of metabolic and apoptosis genes in HT29 cells with FoxO3a knockdown sequences or negative control (NC). (**C**, **D**) IHC analysis of c-MYC expression in cetuximab sensitive and resistant CRC tissues.(**E**) Western blotting analysis of c-Myc protein levels in CRC cells, Caco2, HT29 and Colo205. (**F**) Western blotting analysis of c-Myc protein levels in Caco2-CS and Caco2-CR. (**G**) Western blotting analysis of c-Myc protein levels in 300 μg/ml cetuximab treated HT29 cells. (**H**) Schematic representation of the human c-MYC promoter (c-MYCpWT) and its mutations. Tow putative FOXO-binding elements are shown. -1 indicates the first 5′-nucleotide from the translation initiation site. Underlined nucleotides are mutated sites. (**I**, **J**) Luciferase reporter assay of identification of elements for FoxO3a-induced activation of c-MYC promoter in HT29 cells (I) and Caco2-CR cells (J).

Mitochondria, the key regulator of cell survival through regulating apoptosis and metabolism plays important functions in chemo-resistance. We therefore screened a panel of genes participating mitochondrial related cell metabolism and apoptosis. To our surprise, RT-PCR analysis showed that the metabolism associated genes including aconitase 2 (ACO2), leucyl-tRNAsynthetase 2 (LARS2),mitochondrial ribosomal protein (MRPL12) and pyruvate kinase M2 (PKM2) were significantly down-regulated after FoxO3a knockdown while apoptotic factors like caspase 3, caspase 8, caspase 9, PUMA and NOXA were not altered in both Caco2-CR and HT29 cells (Figure 5A and 5B). And all these metabolic associated genes are mostly regulated by Myc [22], we wondering whether FoxO3a can affect the function of c-Myc. Since the function of FoxO3a is highly context dependent [23], we examined the potential association between FoxO3a and c-Myc. As shown the c-myc staining was much stronger in the resistant tissues than in the sensitive tissues (Figure 5C and 5D).Western blotting analysis of CRC cell lines showed that expression of c-Myc proteins also elevated in HT29 and colo205 cells (Figure 5E) as well as in Caco2-CR cells (Figure 5F). More, c-Myc was significantly elevated after treated with cetuximab (300 μg/ml) in HT29 cells (Figure 5G) indicating that c-Myc changed in consistent with FoxO3a. We then searched the FOXO consensus elements in c-Myc promoter region (TTGTTTAC) and found that two putative elements that resemble the consensus elements (Figure 5H). To assess the functionality of these elements, we generated three mutant (c-MYCp UM, c-MYCp DM, and c-MYCp UDM) in which the respective elements were disrupted (Figure 5H). The luciferase reporter assay demonstrated that FoxO3a-induced enhancement in c-MYC promoter activity was inhibited upon mutation of the downstream putative FOXO binding elements in HT29 and Caco2-CR cells (Figure 5I and 5J). Taken together, these results indicate that FoxO3a activates the c-Myc promoter through directly binding to the FOXO consensus element, thus increasing transcription of c-Myc and subsequent c-Myc downstream metabolic genes.

### Knockdown c-Myc reduced resistance to cetuximab treatment, proliferation and migration in CRC cells

**Figure 6:**
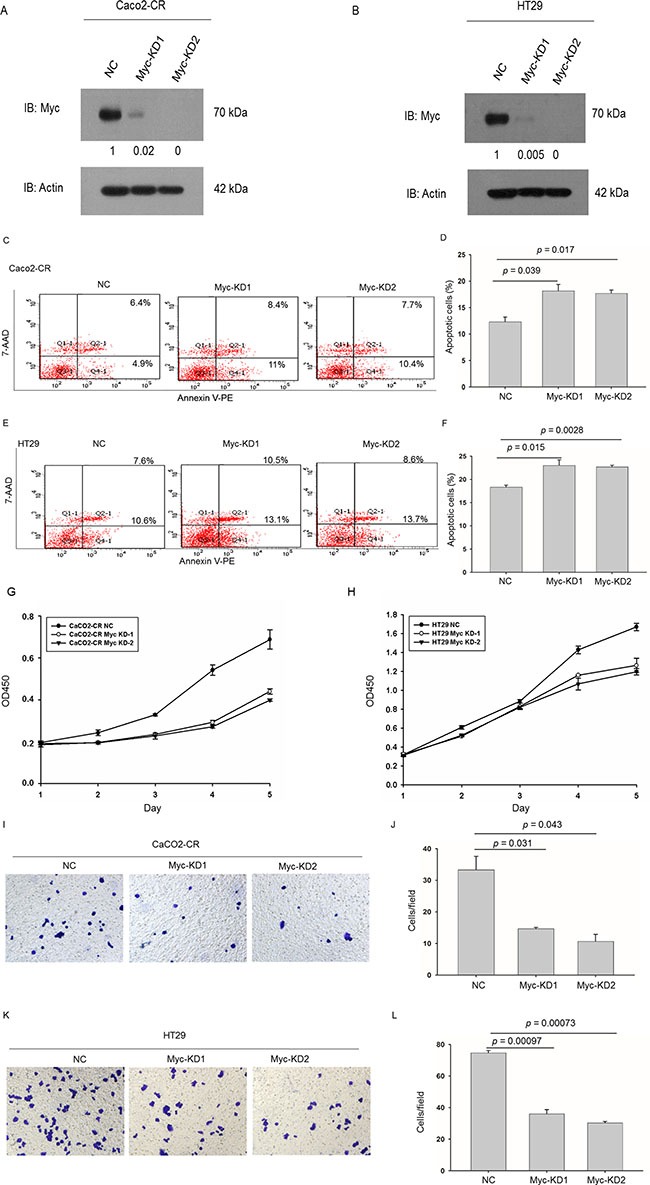
Knockdown of c-Myc sensitized CRC-CR cells to cetuximab treatment and inhibited cell proliferation and migration ability in cetuximab resistant CRC cells (**A**) Western blotting analysis of c-Myc protein levels in Caco2-CR cells with FoxO3a knockdown sequences or negative control (NC). (**B**) Western blotting analysis of c-Myc protein levels in HT29 cells with FoxO3a knockdown sequences or negative control (NC). (**C**, **D**) FACS analysis of cell apoptosis in Caco2-CR cells with or without c-Myc knockdown treated with cetuximab. (**E**, **F**) FACS analysis of cell apoptosis in HT29 cells with or without c-Myc knockdown treated with cetuximab. (**G**) CCK8 analysis of Caco2-CR cells proliferation ability with c-Myc knockdown sequences or negative control (NC). (**H**) CCK8 analysis of Caco2-CHT29 cells proliferation ability with c-Myc knockdown sequences or negative control (NC). (**I**, **J**) Transwell analysis of cell migration ability in Caco2-CR cells with or without c-Myc knockdown. (**K**, **L**) Transwell analysis of cell migration ability in HT29 cells with or without c-Myc knockdown.

**Figure 7:**
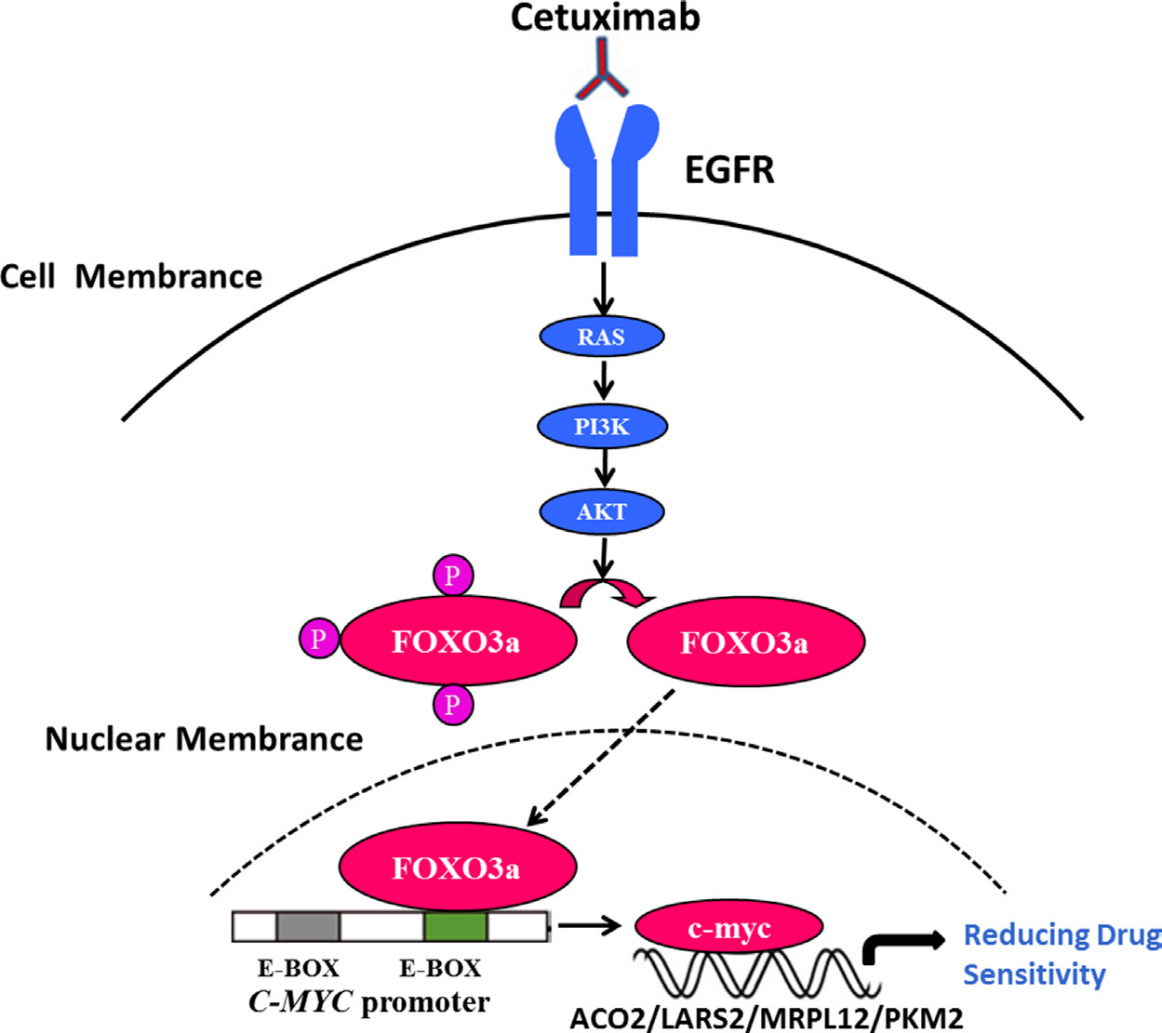
FoxO3a regulated c-Myc was important in cetuximab resistant cell proliferation, migration and survival in CRC Under the consistent stimulation of cetuximab in RAS wild-type mCRC, the up-regulation of FoxO3a in nucleus could directly interact with c-Myc and subsequently activate the downstream metabolic related molecule, leading to the resistance.

To further verify the function of c-Myc in cetuximab resistance, we knocked down c-Myc in Caco2-CR and HT29 cells. Western blot verified the efficient knockdown of c-Myc in Caco2-CR and HT29 cells (Figure 6A and 6B). Consistently, knockdown of c-Myc increased cell apoptosis after cetuximab treatment (Figure 6C–6F). Further, CCK8 analysis showed that c-Myc knockdown reduced cell proliferation in Caco2-CR and HT29 cells (Figure 6G and 6H). Transwell analysis also indicated that inhibition of c-Myc suppressed cell migration in Caco2-CR and HT29 cells (Figure 6I–6L). Altogether, our data revealed that FoxO3a regulated c-Myc was important in cetuximab resistant cell proliferation, migration and survival in mCRC (Figure 7).

## DISCUSSION

In the present study, we found significant increased FoxO3a in cetuximab resistant CRC tissues and cells. Moreover, new mechanism is revealed that FoxO3a regulates metabolic genes through directly activate c-Myc transcription in cetuximab resistant colorectal cancer cells, which confers cetuximab resistance. These findings might help understanding the complicated pathological function and mechanism of FoxO3a in RAS wild-type mCRC resistant to cetuximab and offer new therapeutic target for mCRC treatment.

PI3K/Akt/ FoxO3a is the classical signal pathway downstream of EGFR. Studies have showed that expression of FoxO3a is elevated under stress conditions, including oxidative stress, serum deprivation, and hypoxia [10, 13, 19, 24]. Moreover, Satoru el at. showed that IGF1-AKT-FoxO3a pathway is critical to the development of radiotherapy resistance in glioma cells, and suppression of FoxO3a enhanced response to radiotherapy [17]; Smita el at. comfirmed that lapatinib-induced FoxO transcription factors upregulated c-Myc epigenetically in cooperation with a cascade of epigenetic regulators associated with MLL2 to inhibit sensitivity of the breast cancer cells to lapatinib [25]. Taken together, these findings suggest that FoxO3a may be a potential factor in the development of drug resistance.

Consistent with these reports, we found that the expression of FoxO3a in CRC cells was increased significantly in response to cetuximab treatment at both mRNA and protein levels. We analyzed the potential in preliminary small mCRC samples and found that FoxO3a was highly expressed in cetuximab resistant mCRC tissues. These results suggest that the high expression of FoxO3a might be a predictive biomarker to cetuximab in mCRC. Meanwhile, FoxO3a is regulated by pathways like Akt and AMPK in cancer [26, 27]. Whether these upstream factors, phosphatase or ubiquitinase changed during stresses conditions including chemo-resistance increasing FoxO3a level are unknown.

c-Myc is a key regulator in mCRC regulating cell survival , metabolism [28, 29]. FoxO3a has been reported associated with c-Myc to promote metabolic adaptation to hypoxia [21]. In our study, we found that FoxO3a interaction with c-Myc promoter in cetuximab resistant CRC cells which is consistent with the development of resistance to lapatinib in breast cancer [25]. Altered metabolism in cancer cells is suspected to contribute to drug resistance, and c-Myc plays the key role in the process of altered metabolism characterized by enhanced glycolysis and oxidative phosphorylation and elevated fatty acid and nucleotide synthesis by regulating the downstream metabolic genes [30–33]. Meanwhile, We have found that FoxO3a interaction with c-Myc promoter and regulating metabolic genes as ACO2, LARS2, MRPL12 and PKM2 in cetuximab resistant CRC cells.

However, the metabolic changes regulated by FoxO3a and c-Myc in mCRC cetuximab resistance and genes downstream of c-Myc actually regulated by FoxO3a need further investigation. Meanwhile, targeting FoxO3a/c-Myc pathway or inhibiting metabolic changes might overcome the resistance of mCRC to cetuximab.

Moreover, the precise function of FoxO3a in cetuximab resistance need further studies. Watkins and colleagues identify that FoxO3a enables plasmacytoid DCs to induce tolerance in tumor antigen-specific CD8+T cells [34], indicating that FoxO3a is associated with tumor immune-suppressive microenvironment. Besides chemo-resistance, FoxO3a was found promote invasion and metastasis of cancer. Therefore, fully understanding the function and molecular mechanisms of FoxO3a in a specific tumor type is extremely important when it is employed as a potential target for cancer therapy.

In summary, our findings determined the crucial function of FoxO3a in mCRC cetuximab resistance through regulating of c-Myc downstream metabolic associated genes, suggesting that FoxO3a might be a novel biomarker and therapeutic target in colorectal cancer therapy.

## MATERIALS AND METHODS

### Patients and tissue samples

Our study was approved by the Ethics Committee of Zhong Shan Hospital, Fu Dan University. Written consent was obtained from patients or guardians on behalf of the minor enrolled in the study. 42 patients with histologically confirmed mCRC with RAS wild-type were recruited in this study from January 2011 to December 2013. Initial tissue samples were collected before treatment. 32 patients had cetuximab treatment. After a median follow-up time of 16 months, 14 patients were sensitive to cetuximab which showed partial response (PR) according to Recist 1.1criteria. Of the 14 sensitive patients, secondary resistance occurred in 8 patients after treatment for 6 to 10 months. 6 patients were primary resistant to cetuximab who presented progression disease (PD) at first. 9 patients had stable disease and 3 patients early terminated cetuximab treatment. Furthermore, fresh frozen samples and paraffin embedded samples were collected before the treatment and after secondary resistance in 3 cetuximab sensitive patients. These samples were analyzed for further FOXO3a and c-Myc expression.

### Immunohistochemistry

Slides were routinely de-paraffinizated and rehydrated. The monoclonal antibody against FoxO3a (1:100 dilutions, Cell signaling technology, USA) was used as primary antibody. For antigen retrieval, the slides were heated at 98°C in an citrate buffer (pH 9.0) for 20 mins and cooled naturally to room temperature. Sections were incubated in 0.3% hydrogen peroxide for 20 mins to inactivate endogenous peroxides. The sections were blocked with 5% normal horse serum in PBS for 30 mins and then incubated with the primary antibody overnight at 4°C, then stained using a highly sensitive streptavidin-biotin-peroxidase detection system and counterstained with hematoxylin. A negative control was also incorporated using pre-immune IgG instead of the primary antibody.

### Cell culture

Caco2, HT29 and colo205 cell lines were obtained from Cell Bank of the Chinese Academy of Sciences (Shanghai, China). The above cell lines passed the conventional tests of cell line quality control methods (e.g. morphology, isoenzymes, mycoplasma) and have been conserved in that bank. Last year, the cell lines passed the test of DNA profiling (STR). Caco2 cells were cultured in Dulbecco's Modified Eagle's Medium (HyClone, Logan, UT) containing 10% FBS, while HT29 and colo205 cells were maintained in RMPI-1640 medium (HyClone, Logan, UT) supplemented with 10% FBS in a 5% CO_2_humidified atmosphere at 37°C.

### Cetuximab resistant cells establishment

For a period of 6 months Caco2 cells were continuously exposed to increasing concentrations of cetuximab. The starting dose was the dose causing the inhibition of 50% of cancer cell growth (IC50). The drug dose was progressively increased to 10 μg/mL in approximately 2 months, to 50 μg/mL after other 2 months, and finally, to 300 μg/mL after additional 2 months. The established cetuximab-resistant Caco2 cancer cell line (Caco2-CR) was then maintained in continuous culture with this maximally achieved dose of cetuximab that allowed cellular proliferation.

### Western blot analysis

Cell samples were collected and lysed in RIPA buffer (150 mmol/L sodium chloride, 0.5% sodium deoxycholate, 9.1 mmol/L dibasic sodium phosphate, 1.7 mmol/L monobasic sodium phosphate, 1% Nonidet P-40, 0.1% sodium dodecylsulfate(pH adjusted to 7.6) containing fresh protease inhibitor cocktails (Sigma-Aldrich, St. Louis, MO, USA).

Lysates were cleared by centrifugation. Then, equal amounts of protein were separated on an 10% SDS-Tris/glycine gel using mini-PROTEIN 3 Gel electrophoresis system (Bio-Rad, USA). After that, proteins were transferred to Polyvinylidene Fluoride Membrane (Millipore,USA),and the membrane was blocked with 5% milk power in TBS plus 1% Tween 20. The membranes were incubated with FoxO3aantibody (12829, Cell signaling technology, USA), c-Myc antibody (sc-40, Santa Cruz Biotech, USA) or actin antibody (PAB0174,Abnova,USA) overnight at 4°C. Immune complexes were detected with HRP-conjugated secondary antibodies and visualized by chemiluminescence reagents (ECL, Amersham Biosciences).

### Lentiviral vector mediated FoxO3a-knockdownstable cells

The target sequences for FoxO3a siRNAs were 5′-GCTCTTGGTGGATCATCAA-3′ (FoxO3a-KD1) and 5′-GCATGTTCAATGGGAGCTTGGA-3′ (FoxO3a-KD2). After 48 h, the efficiency of FoxO3a knockdown was confirmed via quantitative real-time polymerase chain reaction (qRT-PCR) and Western blot.

Lentiviral vectors for human FoxO3a-shRNA carrying a green fluorescent protein (GFP) sequence were constructed by Hanyin Co. (Shanghai, China). The recombinant FoxO3a knockdown lentivirus and the negative control (NC) lentivirus (GFP-lentivirus; Hanyin Co. Shanghai,China) were prepared and titered to 10^9^ TU/ml (transfection unit). To obtain the stable FoxO3a-knockdown cell line, Caco2-CR or HT29 cellswere seeded in six-well dishes at a density of 2 × 10^5^ cells per well. Thecells were then infected with the same titer virus with 8 μg/ml polybrene on the following day. Approximately 72 h after viral infection, GFP expression was confirmed under a fluorescence microscope, and the culture medium was replaced with selection medium containing 4 μg/ml puromycin. The cells were then cultured for at least 14 days. The puromycin-resistant cell clones were isolated, amplified in medium containing 2 μg/ml puromycin for seven to nine days, and transferred to a medium without puromycin. The clones were designated as FoxO3a-KD or NC cells.

### Cell proliferation assay

A CCK8 assay was conducted according to the kit instructions (DH343-2, Beijing Dongsheng Biotechnology, China). Briefly, cells were plated at equal cell density (2,000 cell/100 μl perwell) in 96-well plates with cetuximab (300 μg/mL) for continuous detection over a 5-day period. At the beginning of the second day, the culture was terminated by adding 10 μl CCK8 (5 mg/ml) to the original culture medium. After 2 h, the plates were measured using a microplate reader (Biotek Elx800, USA). Cell proliferation was measured using OD490 values.

### Transwell analysis

The invasion assay was performed by using a Transwell (No. 3422, Corning, USA). Cells (2 × 10^4^ cells/well) were seeded into the migration chamber containing serum-free DMEM and incubated for 24 hours at 37°C. Cells that migrated to the lower surface of the filters were stained and counted under a light microscope. All assays were performed in triplicate.

### Scratch assay

Cells grown in 6-well plates were artificially injured by scratching across the plate with 200-μl pipette tips. The wound areas were photographed at 0 and 24 hours and measured using a caliper. The wound closure percentages were calculated using the following formula: (1-[current wound size/initial wound size])*100.

### Cell apoptosis assay

Cells were treated as indicated concentration of cetuximab or irinotecan combination for 24 hours. The Annexin V-PE Apoptosis Detection Kit (catalog# 559763, BD Pharmingen, USA) was applied to assess apoptosis according to the manufacturer's instructions. Briefly, cells were resuspended in 1x Binding Buffer at a concentration of 1 × 10^6^ cells/ml, and 100 μl of this suspension was added to each of the following tubes: (1) an empty tube, (2) a tube containing Annexin V-PE reagent (5 μl); (3) a tube containing a 7-AAD reagent (5 μl); and (4) a tube containing both Annexin V-PE reagent (5 μl) and a 7-AAD reagent (5 μl). Gently vortex the tubes and incubated for 15 min at room temperature in the dark. 1 × Binding Buffer (400 μl) was added to each tube and analyzed by flow cytometry.

### *In vivo* animal model

All animal experiments were approved by the Animal Research Committee of Zhong Shan Hospital, Fu Dan University. Caco2-CR cells (5 × 10^6^ per mouse) with or without FoxO3a knockdown were injected into the subcutaneous of 6–8-week-old Nude mice. The time for tumor growth was about three months. Once palpable, tumors were measured every week and volumes were calculated using formula: a*b2/2 [the largest (a) and the smallest (b)]. After three months, all mice were euthanized using CO2, and tumor tissues were removed and weighted. Every group included 6–8 mice and 3 replicates. All animal studies were approved by the Institutional Animal Care and Use Committee of the Shanghai Institutes for Biological Sciences.

### Promoter assay

A reporter vector containing the human c-MYC promoter (−2000 to +1) was cloned. Two putative FOXO binding elements in the c-MYC promoter region (−1797 to −1790 and −330 to −323) were mutated from TTGTTTTC to TCCCCTTC and CTGTTTAC to CCCCCTAC by site-directed mutagenesis. HT29 or CaCO2-CR (2.5 × 105 cells) cells were seeded onto a 24-well dish and, the next day, were transfected with the reporter and effector constructs using the Fugene HD reagent according to the manufacturer's protocol. After 48 h, a luciferase assay was performed using the Dual-Luciferase Reporter Assay System (Promega).

### Statistical analysis

Triplicate samples were analyzed for each experiment, and two-tailed Student's *t* test was used to analyze the differences between groups using GraphPad Prism 5 (GraphPad Software, SanDiego, CA). *P-*value of < 0.05 was considered statistically significant.

## SUPPLEMENTARY MATERIALS


