# Supplementary material for: FoxO3a confers cetuximab resistance in RAS wild-type metastatic colorectal cancer through c-Myc
Source: Oncotarget. 2016 Nov 4;7(49):80888–900. doi: 10.18632/oncotarget.13105 (PMC5348362; doi:10.18632/oncotarget.13105)
Supplement: Supplementary file 1 [file oncotarget-07-80888-s001.pdf]

FoxO3a confers cetuximab resistance in RAS wild-type metastatic colorectal cancer through c-Myc

Supplementary Materials

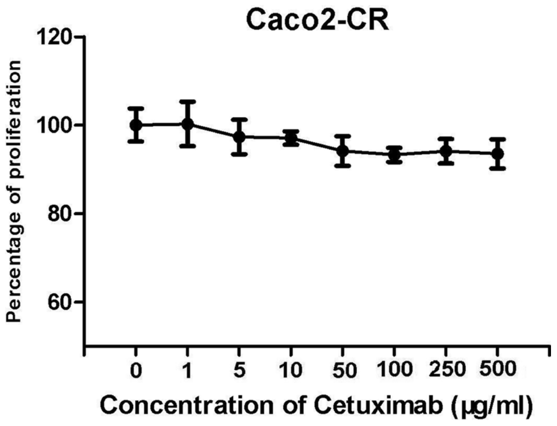

Supplementary Figure S1: CCK8 analysis of the growth curve of resistant cells.
